# Targeting c-MET for Endoscopic Detection of Dysplastic Lesions within Barrett’s Esophagus Using EMI-137 Fluorescence Imaging

**DOI:** 10.1158/1078-0432.CCR-24-1522

**Published:** 2024-11-08

**Authors:** Yi-Jhih Huang, Jonas Rieder, Kel Vin Tan, Anna Tenditnaya, Borivoj Vojnovic, Dimitris Gorpas, Michael Quante, Katherine A. Vallis

**Affiliations:** 1 Department of Oncology, University of Oxford, Oxford, United Kingdom.; 2 Division of Thoracic Surgery, Department of Surgery, Tri-Service General Hospital, National Defense Medical Center, Taipei, Republic of China.; 3 Department of Internal Medicine II, Universitätsklinikum Freiburg, Universität Freiburg, Freiburg, Germany.; 4 Invicro, London, United Kingdom.; 5 Institute of Biological and Medical Imaging, Helmholtz Zentrum München, Neuherberg, Germany.; 6 Chair of Biological Imaging, Central Institute for Translational Cancer Research (TranslaTUM), School of Medicine and Health, Technical University of Munich, Munich, Germany.

## Abstract

**Purpose:**

Esophageal cancer carries a poor prognosis with a 5-year overall survival of less than 20%. Barrett’s esophagus increases the risk of esophageal adenocarcinoma. The aim of this study was to investigate the ability of EMI-137, a mesenchymal–epithelial transition factor (c-MET)-targeting optical imaging tracer, to detect dysplasia in Barrett’s esophagus.

**Experimental Design:**

c-MET expression in human esophageal tissue was investigated using Gene Expression Omnibus datasets, tissue microarrays, and Barrett’s esophagus biopsies. EMI-137 was tested in a dual xenograft mouse model bearing OE33 (c-MET high expression) and FLO-1 (c-MET low expression) tumors. Fluorescence molecular endoscopy was performed in a mouse model of Barrett’s-like metaplasia and dysplasia (L2-IL1β). Tumors and organs of interest were evaluated through *ex vivo* fluorescence imaging.

**Results:**

*MET* mRNA expression analyses and c-MET immunostaining confirmed upregulation of c-MET in Barrett’s esophagus and esophageal adenocarcinoma compared with normal epithelium. There was strong accumulation of EMI-137 in OE33 xenografts 3 hours after injection, decreasing by more than 50% on coinjection of a 10-fold molar excess of unlabeled EMI-137. The target-to-background ratio at 3 hours after injection for OE33 and FLO-1 tumors was 10.08 and 1.42, respectively. Fluorescence molecular endoscopy of L2-IL1β mice showed uptake of EMI-137 in dysplastic lesions within Barrett’s esophagus with a target-to-background ratio of 1.9 *in vivo* and greater than 2 in *ex vivo* fluorescence imaging.

**Conclusions:**

EMI-137 accumulates in dysplastic lesions within Barrett’s esophagus and also in c-MET–positive esophageal adenocarcinoma. EMI-137 imaging has potential as a screening and surveillance tool for patients with Barrett’s esophagus and as a means to detecting dysplasia and esophageal adenocarcinoma.

**Translational Relevance**

The accurate and prompt diagnosis of dysplasia in Barrett’s Esophagus is critically important in the effort to reduce the morbidity and mortality of esophageal adenocarcinoma. Standard practice currently involves the performance of endoscopic examination with random biopsies taken in areas of suspected dysplastic Barrett’s esophagus. In this study, we showed that the transmembrane tyrosine kinase, mesenchymal–epithelial transition factor (c-MET), is overexpressed in dysplastic Barrett’s esophagus tissue. We investigated the use of a systemically delivered c-MET–binding peptide, EMI-137, that is tagged with a near-IR fluorophore, in combination with real-time, high-resolution fluorescence molecular endoscopy (FME). This approach was tested in a transgenic mouse model that phenocopies Barrett’s esophagus and dysplasia, L2-IL1β. EMI-137 accumulated in dysplastic Barrett’s esophagus lesions sufficiently for rapid and facile identification using FME. c-MET–based FME has translational potential as an accurate screening and surveillance tool for patients with Barrett’s esophagus.

## Introduction

The global incidence of esophageal cancer is increasing, with 0.6 million new cases reported in 2020 and with more than half a million deaths attributed to esophageal cancer annually (1, 2). The prognosis of esophageal cancer is unfavorable, although advances in the diagnosis and treatment of this disease are gradually contributing to improved outcomes. A large retrospective cohort study in the United States revealed that there was a significant increase in overall median survival (6–10 months; *P* < 0.001) and the 5-year survival rate (9% to 22%; *P* < 0.001) when comparing 1973–1980 to 2001–2009 (3). Targeted surveillance programs for high-risk individuals, such as those with Barrett’s esophagus, and a growing awareness of the risk factors that may lead to esophageal cancer improve the likelihood of detection at an early, treatable, or even potentially curable stage. Unfortunately, esophageal cancer is currently rarely amenable to curative treatment at diagnosis due to nonspecific symptoms in the early stages of the disease (4, 5).

Barrett’s esophagus is a widely recognized predisposing factor and precursor of esophageal adenocarcinoma. Both Barrett’s esophagus and esophageal adenocarcinoma are more common in males than in females, with ratios in North America of about 3 and 7.6, respectively. The precise pathogenesis and the mechanisms governing disease progression from Barrett’s esophagus to low-grade dysplasia (LGD) or high-grade dysplasia (HGD) and cancer are still under investigation and remain the subject of ongoing research (4, 6–8). The annual progression rate (APR) from Barrett’s esophagus to esophageal adenocarcinoma is variable. In cases of nondysplastic Barrett’s esophagus (NDBE), the APR typically ranges from 0.1% to 0.5%. For individuals with HGD, the APR is >20%. Although several screening tools and programs have been investigated, endoscopy-guided biopsy remains the definitive diagnostic modality for the detection of dysplasia and esophageal adenocarcinoma. The current standard of care, Barrett’s esophagus surveillance, involves the performance of random four-quadrant biopsies of the columnar Barrett’s esophagus epithelium at 2 cm intervals, aided by narrow-band imaging which utilizes blue (400–430 nm) and green (530–550 nm) light filters to identify irregular vascular or mucosal patterns indicative of dysplasia (9, 10). However, these nonspecific approaches are not only invasive but also lack molecular specificity (4, 8). In fact, 20% to 25% of esophageal adenocarcinoma cases are missed during standard endoscopic Barrett’s esophagus surveillance (5). Consequently, identification of currently undetected early dysplastic lesions during endoscopy remains a pivotal issue that must be solved to improve the detection rate of patients with high-risk Barrett’s esophagus and, ultimately, survival from esophageal adenocarcinoma.

Fluorescence molecular endoscopy (FME) is a medical technique that combines white light endoscopy (WLE) with administration of a fluorophore-labeled probe that binds to and visualizes one or more molecular targets (11–16). This technology enables real-time, high-resolution imaging during endoscopic procedures, which allows “red flag” biopsies of areas suspicious for dysplasia rather than random biopsies. FME is also of interest for the early detection of esophageal or colorectal cancer (16, 17), guiding minimally invasive surgery (18, 19), and providing insights into the pathophysiology of cancer and gastrointestinal disorders (20).

EMI-137 is a 26 amino acid peptide that binds the cell-surface receptor, hepatocyte growth factor receptor (also known as c-MET, mesenchymal–epithelial transition factor), that is upregulated in several cancer types, including thyroid, hepatocellular, colorectal, and gastroesophageal cancers (21–25). EMI-137, which incorporates a near-IR dye, Cy5**, has been tested as a noninvasive imaging tracer and, in recent reports, has been tested for intraoperative cancer identification (18, 19) and colorectal polyp recognition (17) and has been piloted in a small group of patients with known esophageal pathology (mainly Barrett’s esophagus with HGD and esophageal adenocarcinoma; ref. 12). However, it remains to be determined whether EMI-137–assisted FME has the potential to serve as a screening tool for Barrett’s esophagus, esophageal dysplasia, or early esophageal adenocarcinoma detection in high-risk populations. The primary aim of this preclinical study was to investigate the feasibility of using EMI-137 in conjunction with FME as a screening approach for Barrett’s esophagus.

## Materials and Methods

### Gene Expression Omnibus dataset analyses

Two independent Gene Expression Omnibus (GEO) datasets (RRID: SCR_005012, GSE26886, and GSE13898) were analyzed (26, 27). The GPL570 Affymetrix Human Genome U133 Plus 2.0 Array platform was used to generate GSE26886 which contains sequencing data from 19 normal squamous epithelium (NSE), 20 Barrett’s esophagus, and 21 esophageal adenocarcinoma samples. The GPL6102 Illumina Human-6 v2.0 Expression BeadChip platform was used to generate GSE13898 which contains gene expression profiles of 28 NSE, 15 Barrett’s esophagus, and 64 esophageal adenocarcinoma samples. The data were normalized to avoid heterogeneity and analyzed using publicly available GEO2R tools (https://www.ncbi.nlm.nih.gov/geo/geo2r/; ref. 28).

### Tissue microarrays, Barrett’s esophagus biopsy samples, and IHC

Human tissue microarrays (TMA) were obtained from Insight Biotechnology (catalog #ES803, ES804, ES809, and ES8011b, US Biomax) and analyzed following ethical review by the Medical Sciences Interdivisional Research Ethics Committee, Oxford University (Reference: R79919/RE003). The human tissue samples included in the TMAs were collected with written informed consent from the donors. Following deparaffinization and rehydration, the TMAs were immersed in preheated 10 mmol/L sodium citrate buffer with 0.05% Tween-20, pH 6.0, at 100°C for 20 minutes for antigen retrieval and then allowed to cool to room temperature. Immunostaining was performed using a primary anti–c-MET antibody (EP1454Y, working dilution: 1:200 for human tissue and 1:1,600 for L2-IL1β mouse tissue; Abcam, Cat. # ab51067; RRID: AB_880695). Slides were counterstained using hematoxylin (Abcam) and allowed to air-dry overnight. Slides were treated with DPX mounting medium (Sigma-Aldrich), and coverslips were applied prior to scanning. Endoscopic human Barrett’s esophagus biopsy samples were provided by Dr E. Bird-Lieberman, Department of Gastroenterology, Oxford University Hospitals NHS Foundation Trust, and stored under license HTA12217. Tissue samples were embedded in optimal cutting temperature compound (OCT) and snap-frozen in liquid nitrogen for 1 to 2 minutes. Sections (5 μm) were prepared using a Cryostat (Leica) and fixed immediately by immersion in ice-cold acetone for 5 to 10 minutes. Slides were air-dried for 5 minutes and stained immediately or stored at −80°C. All digital images were acquired using an Aperio CS2 pathology slide scanner (Leica). Irrelevant specimens included in the TMAs, such as squamous cell carcinoma and small cell carcinoma, were excluded from subsequent analyses and only NSE, esophageal inflammation, hyperplasia, and esophageal adenocarcinoma samples were included. IHC scores (*H*-scores) were calculated using the QuPath deep learning pathology software version 0.4.3 by multiplying the percentage of positive cells (P) by staining intensity (1+, weak; 2+, moderate; and 3+, strong), giving *H*-score = 1 × P_1+_ + 2 × P_2+_ + 3 × P_3+_ and an *H*-score range of 0 to 300 (29). All the cells in each TMA core were included in these analyses. Positivity of staining was defined as the number of positively stained cells in a core section divided by the total number of cells in the section.

### Cell culture

Human esophageal adenocarcinoma cell lines, OE33 (RRID: CVCL_0471, high c-MET expression, obtained from Sigma-Aldrich, female sex), and FLO-1 (RRID: CVCL_2045, low c-MET expression, obtained from the ATCC, male sex), were grown in RPMI 1640 or DMEM medium, respectively, and maintained at 37°C in 5% CO_2_ in a humidified incubator. The level of c-MET expression in OE33 and FLO-1 cells was confirmed by IHC and immunofluorescence microscopy (Supplementary Fig. S1A–S1H). *Mycoplasma* testing was performed every 6 to 8 weeks using a MycoAlert detection kit (Lonza). Cell authentication was performed by short tandem repeat analysis (NorthGene; most recently on April 29, 2024).

### EMI-137 binding affinity and competitive binding assays

OE33 and FLO-1 were seeded (5 × 10^4^ cells per well) in 96-well plates (Greiner Bio-One) and incubated overnight to allow cell attachment. In saturation binding assays, EMI-137 was added to OE33 and FLO-1 in a range of concentrations (0–32 nmol/L) and incubated for 2 hours. For competitive binding assays, EMI-137 (10 nmol/L) and unlabeled EMI-137 (0.39–1,000 nmol/L) were mixed, added to OE33, and incubated for 2 hours. After incubation, cells were washed three times with phenol red–free RPMI 1640 (Thermo Fisher Scientific) or FluoroBrite DMEM medium (Thermo Fisher Scientific). The fluorescence intensities were recorded immediately using an Infinite M200 Pro multimodal plate reader (Tecan).

### Live cell confocal microscopy

FLO-1 and OE33 cells were seeded onto sterile chamber slides (Thistle Scientific) at a density of 2 × 10^4^ cells per well, allowed to adhere overnight, and then incubated in fresh medium containing either EMI-137 (10 nmol/L) or a mixture of EMI-137 (10 nmol/L) plus unlabeled EMI-137 (100 nmol/L) for 2 hours. Following incubation, the cells were washed three times using phenol red–free medium. Nuclei were counterstained with Hoechst 33342 dye (Thermo Fisher Scientific). Live cell images were acquired using a LSM 880 confocal microscope (Carl Zeiss).

### Dual xenograft model, *in vivo* imaging, and *ex vivo* biodistribution

Animal procedures were performed in accordance with the UK Animals (Scientific Procedures) Act 1986 and with local Animal Welfare and Ethical Review Board approval. Six-week-old, female, NOD/SCID gamma mice were used (Charles River Laboratories, RRID: IMSR_JAX:005557). To evaluate the extent of EMI-137 accumulation in c-MET-expressing esophageal adenocarcinoma tumors *in vivo*, a dual xenograft model was established (Supplementary Fig. S2A), which reduced the total number of animals needed for the experiment and ensured that for each mouse, exposure to EMI-137 was identical for both c-MET–positive and –negative tumors. Mice were housed in individually ventilated cages in an artificial 12-hour day and night cycle with access to water and food *ad libitum*. OE33 and FLO-1 cells were washed three times with serum-free medium and mixed in a 1:1 volume with Cultrex Basement Membrane Extract (BME) type 3 matrix (Bio-Techne) to the desired concentration. Notably, the growth rate of FLO-1 tumors is faster than that of OE33 (Supplementary Fig. S2B). Therefore, FLO-1 cells were inoculated 7 to 10 days after OE33 so that the tumors were of similar size at the start of experiments. Mice were anesthetized using isoflurane inhalation (2% v/v isoflurane), and OE33 (5 × 10^6^ cells, 100 μL, right flank) and FLO-1 (2 × 10^6^ cells, 100 μL, left flank) were inoculated subcutaneously. Mice were entered into study when tumor volume reached between 100 and 300 mm^3^. OE33 xenograft tissue was immunostained for c-MET (Supplementary Fig. S2C). Prior to administration of EMI-137, an alfalfa-free rodent diet (Envigo) was used for at least 7 days to reduce autofluorescence originating from standard chow. EMI-137 (0.25 mg/kg) was injected intravenously via a lateral tail vein. Following induction of general anesthesia, mice were placed in an *in vivo* imaging system (IVIS, PerkinElmer). Images were acquired using excitation and emission wavelengths of 640 and 680 nm, respectively. The field of view was set to 12.5 cm to ensure inclusion of the whole animal. For serial imaging, IVIS fluorescence images were acquired every 20 minutes for up to 160 minutes post injection (p.i.; Supplementary Fig. S3A and S3B). To investigate the biodistribution of EMI-137 in the dual xenograft model, organs were collected and imaged *ex vivo* after euthanasia. After image acquisition, radiance efficiency and the target-to-background ratio (TBR) were calculated by conducting region-of-interest (ROI) analysis (Living Image software, RRID: SCR_014247, version 4.7, PerkinElmer). The background region was set as non–fur-bearing skin (ear).

### L2-IL1β mouse model of Barrett’s esophagus

FME and IVIS imaging experiments were conducted according to the German Animal Welfare Act (reference number: 35-9,185.81/G-20/175, Germany) and under the approval of the regional authority of Freiburg (Regierungspräsidium Freiburg). The experimental scheme is shown in Supplementary Fig. S4. In the L2-IL1β mouse model, the expression of the human *IL1β* gene in the esophagus and stomach is under the control of Epstein-Barr virus promoter (ED-L2), as previously described (30). Overexpression of IL1β leads to chronic inflammatory esophagitis and progression to metaplasia, similar to human Barrett’s esophagus, in 100% of L2-IL1β mice, and 20% of L2-IL1β mice go on to develop HGD or esophageal adenocarcinoma (30, 31). Histopathologic analysis of lesions that develop in L2-IL1β mice has shown upregulation of trefoil factor 2 (TFF2), Bmp4, Cdx2, Notch1, and IL6, which are esophageal markers typical of human Barrett’s esophagus and dysplasia (30). L2-IL1β mice (RRID: MGI:5308267) were weaned at 21 days, provided with water and standard chow diet (Granovit AG) *ad libitum*, and genotyped at 6 to 8 weeks of age. As the focus of this study was detection of dysplasia, mice between the ages of 4 and 16 months were entered into the study. They were assigned into three groups according to the anticipated severity of Barrett’s esophagus/dysplastic lesions: 4 months old (low score), 7 to 8 months old (intermediate score), and 10 to 16 months old (high score; *n* = 3 per group; *n* = 9 in total). Both male and female mice were used in FME experiments (M:F = 2:7). One additional L2-IL1β mouse per group received PBS intravenously as a negative control. For disease progression assessment, macroscopic scoring was performed. Each stomach and esophagus was evaluated for lesion extent, individual lesion size, total lesion size, and summed for an overall macroscopic score (low, intermediate, or high), as previously described (31, 32). For standardization, lesions were circled using ImageJ (RRID: SCR_003070), and the lesion area was measured; then the length of the cardia was measured as a straight line, and the quotient (lesion area divided by cardia length) was calculated. To ensure minimal solid food remnant in the stomach, the rodent diet was replaced by DietGel (Fa. Ssniff, DietGel Boost high-calorie dietary supplement) and water *ad libitum* for 2 days before the experiment, and DietGel was then withdrawn 6 hours before euthanasia and FME.

### FME of L2- IL1β mice

Two hours prior to FME, mice received either 50 μg EMI-137 diluted in 100 μL PBS intravenously or 100 μL PBS intravenously (negative control). Immediately after euthanasia, the endoscope was inserted orally to image the esophagus and stomach. For every pullback, ROIs corresponding to Barrett’s esophagus–like and dysplasia-like lesions and background areas were defined in selected frames, and the average pixel intensities were quantified. All intensities were normalized by the acquisition parameters (i.e., exposure time and gain, both linear to the signal level) to ensure comparability of signals between pullbacks. For the specific application, the impact of working distance and viewing angle is minimal, as the dimension of the mouse esophagus is very small. For every L2-IL1β mouse, at least three passes of the endoscope were performed, and each time a pullback video was acquired to ensure consistency of the acquired signal. The TBR was calculated from the corresponding signal intensities for each frame in each group. The overlay images are created by merging color and fluorescence images using alpha channel transparency, as previously described (33). A full description of the FME system is provided in Supplementary Material S1.

### IVIS imaging and immunostaining of L2-IL1β tissues

Following endoscopy, mice were euthanized, and the esophagus and stomach were excised and opened to expose the lumen, washed thoroughly with PBS to remove food remnants, and then imaged *ex vivo* using the IVIS imaging system. The anatomy of mouse stomach is shown in Supplementary Fig. S5A and S5B. ROIs were the squamocolumnar junction (the junction between the forestomach and the gastric cardia/corpus) and esophagogastric junction (EGJ), with the corresponding background regions (adjacent normal gastric mucosa in the corpus of the stomach). Afterward, the organs were formalin-fixed, paraffin-embedded for hematoxylin and eosin and c-MET immunostaining. Multiple formalin-fixed, paraffin-embedded sections from each animal were evaluated. Lesions were assigned a score for dysplasia, as described in reference 31: (i) (superficial epithelial atypia), (ii) (atypia in glandular complexity), (iii) (LGD), and (iv) (HGD). The intensity of c-MET immunostaining was scored as follows: 0, negative; 1, mild; 2, moderate; and 3, strong staining.

### Data collection, analysis, and statistics

Data analyses were carried out using GraphPad Prism (RRID: SCR_002798) version 10.0.3 for MacOS. GSE26886 and GSE13898 datasets were available in the GEO database. Other data analyzed in this study are available from the corresponding author on reasonable request. Descriptive and quantitative data are expressed as the mean ± SEM. Categorical and continuous variables were compared using the independent *t* test. Multiple categorical variables were compared by one-way ANOVA. *P* values of less than 0.05 were regarded as statistically significant. Some figures were generated using Bio.Render.com (as indicated in individual figure legends).

### Data availability

GSE26886 and GSE13898 datasets are available in the GEO database. Other data generated in this study are available upon request from the corresponding author.

## Results

### c-MET expression in Barrett’s esophagus and esophageal adenocarcinoma

**Figure 1.**
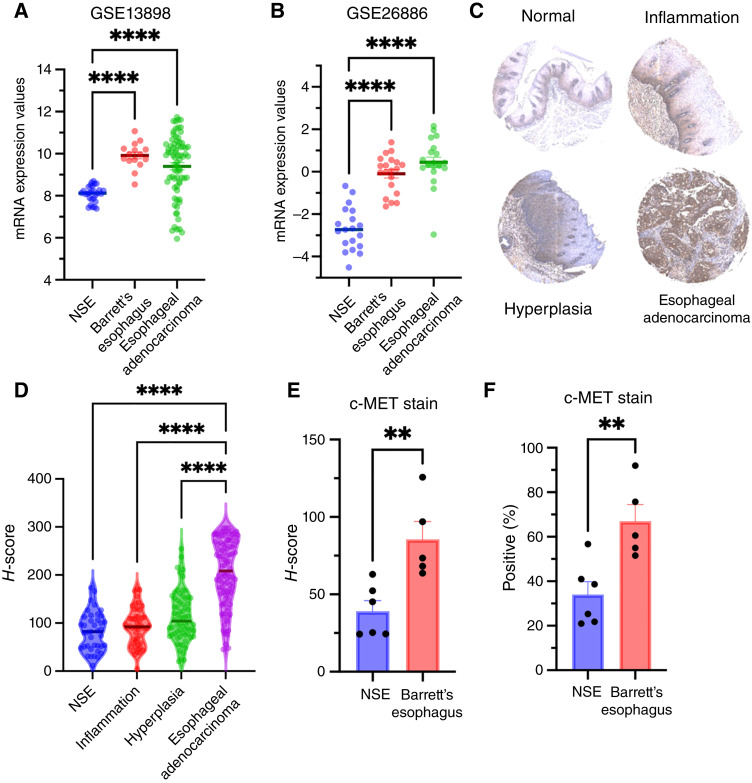
c-MET is upregulated in Barrett’s esophagus and esophageal adenocarcinoma compared with normal epithelium: **A** and **B**, Upregulation of *MET* mRNA in Barrett’s esophagus and esophageal adenocarcinoma in two GEO databases in comparison with NSE. **C,** Representative immunostaining of normal epithelium, inflammation, hyperplasia, and esophageal adenocarcinoma. **D,** Analysis of human TMAs confirmed upregulation of c-MET protein in esophageal adenocarcinoma compared with NSE, inflammation, and hyperplasia (all *P* < 0.0001). **E** and **F,** Immunostaining of endoscopic human biopsy samples of NSE (*n* = 6) and Barrett’s esophagus (*n* = 5) showed an approximately 2-fold greater average H-score and percentage of c-MET positivity in Barrett’s esophagus compared with NSE. **, *P* < 0.01; ****, *P* < 0.0001.

Analysis of the GSE13898 dataset revealed a notable increase in *MET* mRNA expression in both Barrett’s esophagus (9.911 ± 0.160) and esophageal adenocarcinoma (9.398 ± 0.171) compared with NSE (8.080 ± 0.073; *P* < 0.0001; Fig. 1A). Similarly, in the GSE26886 dataset, upregulation of *MET* mRNA expression was observed in both Barrett’s esophagus (−0.096 ± 0.198) and esophageal adenocarcinoma (0.447 ± 0.236) compared with NSE (−2.692 ± 0.235; *P* < 0.0001; Fig. 1B). To confirm whether c-MET protein expression is elevated in Barrett’s esophagus and neoplastic tissue, TMAs and Barrett’s esophagus biopsy samples were analyzed. TMA immunostaining (Fig. 1C) for c-MET revealed *H*-scores of 82.51 ± 6.41 (NSE, *n* = 29), 92.00 ± 5.45 (inflammatory esophageal tissue, *n* = 50), 112.50 ± 6.20 (hyperplasia, *n* = 68), and 205.80 ± 6.74 (esophageal adenocarcinoma, *n* = 104), respectively (Fig. 1D). The *H*-score of esophageal adenocarcinoma is significantly higher than that of NSE, inflammatory, or hyperplastic esophageal tissue (all *P* values < 0.0001). All other comparisons were statistically nonsignificant except that a borderline difference between NSE and hyperplasia was observed (*P* = 0.0384). Clinical metadata associated with the samples represented on the TMAs are presented in Supplementary Tables S1 and S2. To further explore the expression status of c-MET in Barrett’s esophagus, immunostaining of human esophageal biopsy samples (six NSE and five Barrett’s esophagus) was performed. The c-MET *H*-scores for NSE and Barrett’s esophagus were 39.10 ± 6.83 and 84.76 ± 10.86 and the c-MET positivity scores were 34.07 ± 5.72 and 55.00 ± 7.87, respectively. Both the c-MET *H*-score and the percentage of positively stained cells were significantly higher in Barrett’s esophagus samples compared with NSE (all *P* values < 0.01; Fig. 1E and F). Taken together, these data indicate higher expression of *MET* mRNA and c-MET protein in Barrett’s esophagus and esophageal adenocarcinoma compared with NSE and benign conditions (inflammation and hyperplasia).

### EMI-137 affinity, specificity, and live-cell imaging

**Figure 2.**
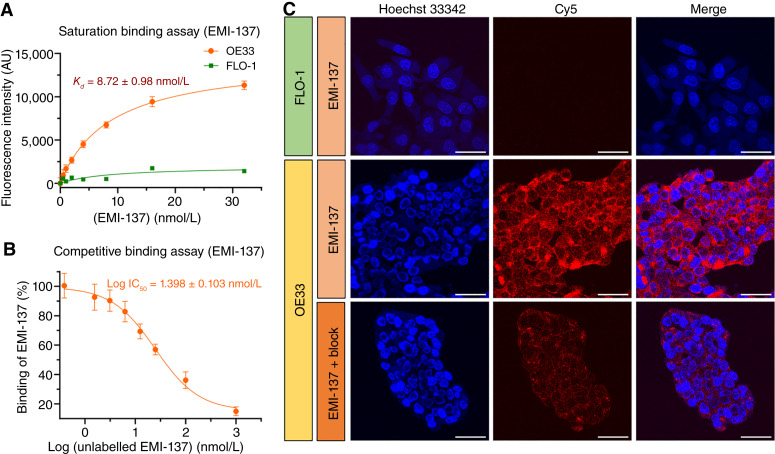
EMI-137 binding affinity and specificity: **A**, Saturation binding assay showing the *K_d_* value of EMI-137 is 8.72 nmol/L. **B,** Competitive binding assay of EMI-137 indicating the IC_50_ value of unlabeled EMI-137 is 25 nmol/L. **C,** Live-cell microscopy FLO-1 cells showing an absence of immunofluorescence staining for c-MET, whereas there is a strong signal in OE33 cells at 2 hours after the addition of EMI-137. Fluorescence in OE33 cells was markedly reduced when cells were exposed to a mixture of 100 nmol/L unlabeled EMI-137 with 10 nmol/L EMI-137. Scale bar, 50 μm. AU, Arbitary unit.

In binding affinity assays, the dissociation constant (*K*_*d*_) of EMI-137 was 8.72 ± 0.98 nmol/L (Fig. 2A). Competitive binding assays showed the IC_50_ value (the inhibitory concentration of 50%) for EMI-137 was 25 nmol/L in OE33 cells (Fig. 2B). Confocal microscopy revealed that FLO-1 cells exhibited minimal fluorescence following exposure to EMI-137, whereas OE33 cells displayed robust fluorescence. Importantly, the fluorescence signal in OE33 cells was almost completely abolished when EMI-137 was applied together with a 10-fold molar excess of unlabeled EMI-137, indicating that the extent of nonspecific binding of EMI-137 is modest (Fig. 2C).

### EMI-137 IVIS imaging in the dual xenograft mouse model

**Figure 3.**
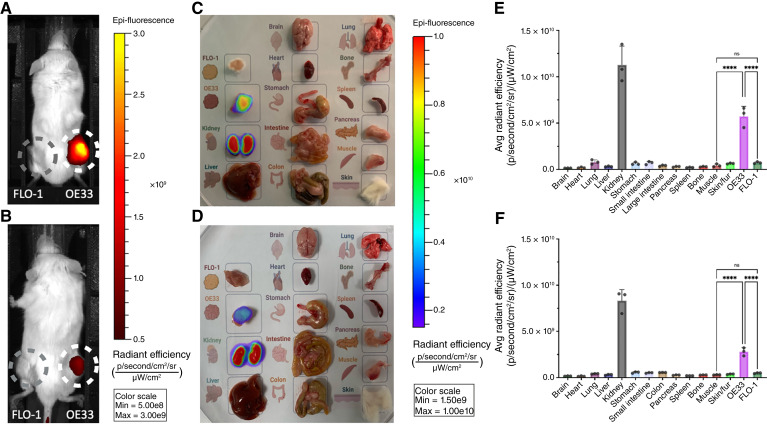
*In vivo* and *ex vivo* fluorescence imaging of EMI-137 in the dual xenograft model: **A,***In vivo* fluorescence imaging 3 hours p.i. of EMI-137 showed a strong signal in the OE33 xenograft (right flank, white circle) but not in the FLO-1 xenograft (left flank, gray circle). **B,** Uptake of EMI-137 in OE33 tumors is effectively but not completely suppressed by coinjecting a 10-fold molar excess of unlabeled EMI-137 as a competitive blocking agent. **C,** Representative *ex vivo* tumor and organ panel of the dual xenograft model 3 hours p.i. of EMI-137 showing high uptake in OE33 tumor and kidney. **D,***Ex vivo* tumor and organ panel taken from mice that received EMI-137 plus unlabeled EMI-137 coinjection as a blocking agent. **E,** Average radiant efficiency in tumors and organs 3 hours p.i. of EMI-137. **F,** Average radiant efficiency in tumors and organs 3 hours p.i. of EMI-137 plus a 10-fold molar excess of unlabeled EMI-137 showed more than 50% reduction of fluorescence in OE33 xenograft compared with **E**. *n* = 3 mice per group. ****, *P* < 0.0001. Avg, average; Max, maximum; Min, minimum; ns, not significant.

Serial *in vivo* fluorescence images acquired at 20-minute intervals following administration of EMI-137 are shown in Supplementary Fig. S3A. In the dual xenograft model, there was a significant accumulation of EMI-137 in OE33 xenografts at 3 hours p.i. In contrast, uptake of EMI-137 in FLO-1 xenografts was minimal at this timepoint (Fig. 3A). The maximum *in vivo* fluorescence intensity in OE33 and FLO-1 tumors was observed at 1 hour p.i. [3.42 ± 1.01 × 10^9^ vs. 0.89 ± 0.29 × 10^9^ (p/second/cm^2^/sr)/(μW/cm^2^); *P* = 0.002; Supplementary Fig. S3B]. The TBR increased from 5.8-fold at 20 minutes p.i. to 10.1-fold at 160 minutes p.i. in OE33 xenografts but did not change in FLO-1 tumors (1.5-fold at 20 minutes and 1.4-fold at 160 minutes). Coinjection of EMI-137 with a 10-fold molar excess of unlabeled EMI-137 resulted in significant reduction of intratumoral fluorescence at 3 hours p.i. in OE33 xenografts compared with administration of EMI-137 alone: 2.59 ± 0.54 × 10^9^ versus 6.53 ± 1.24 × 10^8^ (p/second/cm^2^/sr)/(μW/cm^2^), *P* < 0.001 (Fig. 3B). *Ex vivo* fluorescence imaging of tumors and organs at 3 hours p.i. revealed that EMI-137 is mainly metabolized and excreted by the kidney (Fig. 3C and D). In addition to the uptake of EMI-137 in OE33 and FLO-1 tumors (57.3 ± 6.26 × 10^8^ vs. 7.30 ± 0.56 × 10^8^; *P* < 0.0001), mild uptake was also observed in the lung (8.07 ± 1.38 × 10^8^), small intestine (7.09 ± 0.98 × 10^8^), stomach (6.45 ± 0.82 × 10^8^), large intestine (4.18 ± 0.41 × 10^8^), and liver (3.28 ± 0.26 × 10^8^; Fig. 3E). A 51% decrement in *ex vivo* fluorescence radiant efficiency of OE33 tumors, from 57.3 ± 6.26 × 10^8^ to 28.1 ± 2.50 × 10^8^ (p/second/cm^2^/sr)/(μW/cm^2^), was observed in the presence of 10-fold molar excess of unlabeled EMI-137 (Fig. 3F; *P* < 0.0001, one-way ANOVA with Tukey multiple comparison test). Apart from the colon (4.18 ± 0.41 × 10^8^ vs. 5.61 ± 0.07 × 10^8^; *P* = 0.0262) and skin (6.49 ± 0.29 × 10^8^ vs. 3.78 ± 0.22 × 10^8^; *P* = 0.0018), there were no other significant differences in fluorescence in the organs of the group receiving EMI-137 alone versus the group that received EMI-137 plus unlabeled EMI-137 (independent *t* test; all *P* > 0.05).

### Real-time FME in L2-IL1β mice

**Figure 4.**
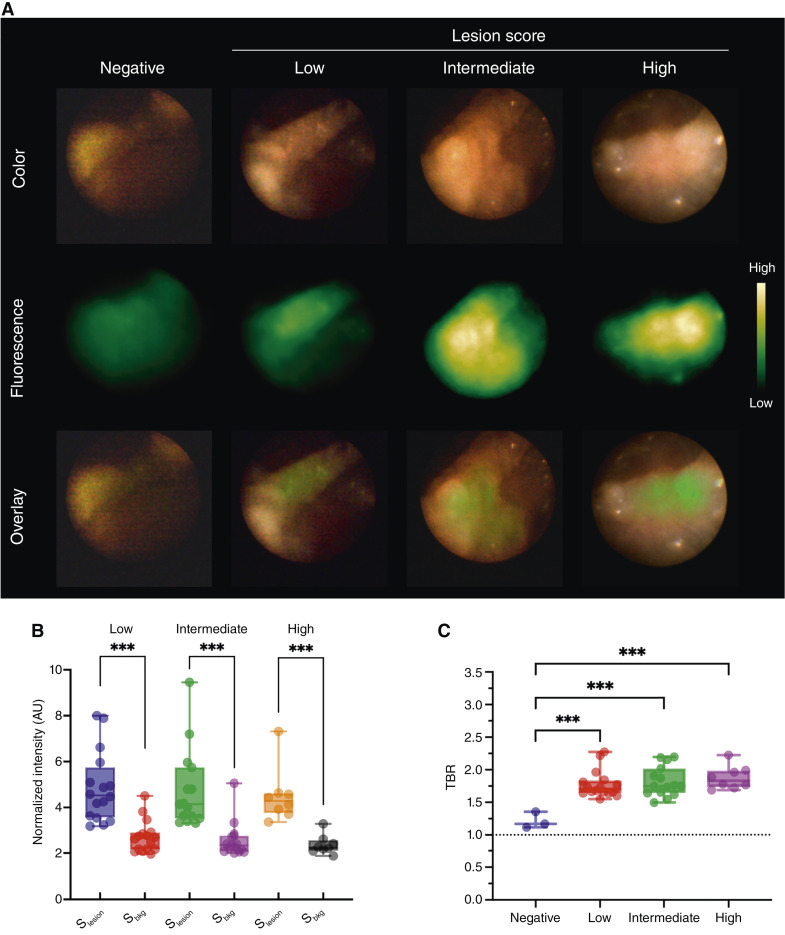
Real-time FME imaging of L2-IL1β mouse after injection of EMI-137: **A,** Representative white-light, fluorescence, and overlay images of L2-IL1β mice with different lesion scores after EMI-137 injection. **B,** Normalized fluorescence signal intensities of the lesion and background in low- (*n* = 16 frames), intermediate- (*n* = 15 frames), and high-score (*n* = 8 frames) groups. **C,** Increased TBR in low-, intermediate- and high-score groups after EMI-137 injection compared with controls receiving PBS. ***, *P* < 0.001. AU, Arbitary unit; S_bkg,_ signal intensities of the background; S_lesion,_ signal intensities of the lesion.

To mimic the endoscopic utilization of EMI-137, we performed endoscopy in L2-IL1ß mice at different disease stages, as previously described (detailed characteristics of the mice are summarized in Table 1; refs. 15, 16). Representative FME videos taken from L2-IL1ß mice with high and low lesion scores are available (Supplementary Videos S1 and S2). Lesions were localized by WLE as well as FME and were assigned macroscopic and histopathologic scores, enabling their categorization to low-, intermediate-, or high-score groups for subsequent statistical evaluation. The average time interval between administration of EMI-137 and FME was 126.4 ± 3.3 minutes (range: 119–152 minutes). Representative, randomly selected FME images (after automated measuring) of lesions with low, intermediate, and high scores following administration of EMI-137 and FME images from control L2-IL1ß mice that received PBS only are shown in Fig. 4A. The top and middle rows display the color and fluorescence images, whereas the bottom row shows an overlay of both. A modest increase in the fluorescence signal was noted in the low-score group, whereas more intense fluorescence was evident in the intermediate- and high-score groups (although these differences did not reach statistical significance). Overlay images demonstrate that the fluorescence signal was localized to the lesions, indicating specific targeting of lesions by the EMI-137 tracer. Normalized fluorescence intensities for the low-, intermediate-, and high-score groups were 4.51 ± 0.43, 4.74 ± 0.45, and 4.87 ± 0.38 arbitary units (AU), respectively, with corresponding background mean fluorescence intensities of 2.38 ± 0.15, 2.58 ± 0.20, and 2.72 ± 0.18 AU (all *P* < 0.001; independent *t* test; Fig. 4B). The average TBR for the PBS-only group was 1.21 ± 0.07, whereas, in comparison, the TBR for the low-, intermediate-, and high-score groups reached 1.78 ± 0.05, 1.82 ± 0.06, and 1.88 ± 0.06, respectively (all *P* < 0.001; independent *t* test; Fig. 4C).

**Table 1.** Details of L2-IL1β *ex vivo* IVIS experiment. Average radiant efficiencies of ROIs were calculated from *ex vivo* IVIS images: left and right squamocolumnar junctions, the junction between the forestomach and the cardiac/corpus of the stomach, and EGJ, with the corresponding background regions located in the corpus of the stomach.

	Mouse profiles			L2-IL1β *ex vivo* IVIS experimental details									
Groups	Gender (M/F)	Age (months)	Lesion score	FME time minutes p.i.	Injectate (i.v.)	Average radiant efficiency × 10^7^ (p/second/cm^2^/sr)/(μW/cm^2^)							
						L_SCJ_	L_bkg_	EGJ	EGJ_bkg_	R_SCJ_	R_bkg_	S_lesion_	S_bkg_
4 months	M	4	Low (45.1)	121	EMI-137	43.19	32.38	45.32	26.75	41.43	23.52	34.50 ± 2.70	22.30 ± 1.68
	F	4	Low (32.8)	121	EMI-137	24.65	16.21	32.22	19.07	35.51	17.13		
	F	4	Low (38.0)	124	EMI-137	25.76	22.50	37.62	19.88	24.61	22.85		
7–8 months	F	7	Intermediate (67.7)	120	EMI-137	103.1	58.93	152.70	64.87	101.5	81.29	91.49 ± 8.74	48.23 ± 6.09
	F	7	Intermediate (64.8)	152	EMI-137	94.01	43.04	84.07	50.36	80.96	46.35		
	F	8	Intermediate (60.9)	148	EMI-137	69.76	24.64	64.50	25.87	81.02	38.71		
10–16 months	F	16	High (93.1)	130	EMI-137	217.4	117.7	266.1	178.7	230.3	164.1	196.9 ± 13.9	102.3 ± 15.5
	F	16	High (88.2)	121	EMI-137	157.0	51.77	152.1	71.54	161.2	66.09		
	M	10	High (76.9)	119	EMI-137	154.9	54.80	227.2	97.49	207.0	117.8		
Control	F	4	Low (38.9)	120	PBS	2.678	1.673	2.125	1.540	2.912	1.911	3.78 ± 0.39	3.35 ± 0.53
	F	7	Intermediate (67.9)	121	PBS	4.740	3.629	2.939	2.832	5.552	4.603		
	F	16	High (97.9)	120	PBS	4.303	3.494	3.800	3.941	5.003	6.496		

Data are presented as average radiant efficiencies [unit: [(p/second/cm^2^/sr)/(μW/cm^2^)], in which p, photons; cm^2^, centimeter square; sr, steradian; and μW, microwatt, unit of the laser power given].

Abbreviations: EGJ, fluorescence of EGJ; EGJ_bkg_, fluorescence of adjacent background of EGJ; L_SCJ_, fluorescence of left SCJ; L_bkg_, fluorescence of adjacent background of left SCJ; Mins, minutes; R_SCJ_, fluorescence of right SCJ; R_bkg_, fluorescence of adjacent background of right SCJ; SJC, squamocolumnar junction.

### Histologic characterization and IVIS imaging

**Figure 5.**
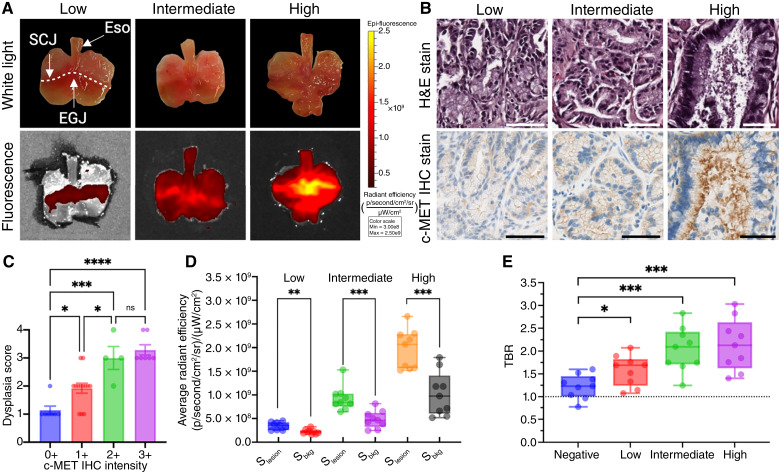
*Ex vivo* fluorescence imaging of L2-IL1β mice: **A,** Representative white light and fluorescence images of the excised stomach taken from L2-IL1β mice. The white dotted line shows the SCJ, which is within the stomach in mice. A protruding, irregular lesion is visible at the EGJ and SCJ in the stomach taken from a high-score group mouse, with the corresponding fluorescence image below. **B,** Upregulation of c-MET in dysplastic lesions from the low-, intermediate-, and high-score groups. **C,** Correlation between dysplasia score and level of c-MET expression. **D,** Box-whisker plot of ROI analysis, which shows increasing fluorescence from low- to intermediate- to high-score lesions. **E,** Quantification of TBR in *ex vivo* IVIS fluorescence imaging. Range of box-whisker plots indicate minimum to maximum. *, *P* < 0.05; **, *P* < 0.01; ***, *P* < 0.001; ****, *P* < 0.0001. Eso, esophagus; H&E, hematoxylin and eosin; Max, maximum; Min, minimum; ns, not significant; S_bkg,_ signal intensities of the background; SJC, squamocolumnar junction; S_lesion,_ signal intensities of the lesion.

Following FME of L2-IL1β mice, stomachs were removed and imaged using white light (Fig. 5A) and correlated with macroscopic and histopathologic scores, confirming the development of Barrett’s esophagus and dysplasia with increasing age, as previously described (30). Average macroscopic lesion scores for low-, intermediate-, and high-score groups were 38.63 ± 3.57, 64.47 ± 1.97, and 86.07 ± 4.80, respectively (one-way ANOVA; *P* = 0.0003). The fluorescence intensity in IVIS images of *ex vivo* stomachs increased with increasing lesion score (Fig. 5A), and the signal localized with abnormal epithelium, consistent with the FME findings. Histopathologic analysis of the Barrett’s esophagus lesions at the EGJ was carried out. Immunostaining revealed increasing expression of c-MET in lesions with an intermediate or high score compared with the low-score group (Fig. 5B). The average c-MET immunostaining intensity score in the dysplasia score groups was 0.33 ± 0.17, 1.00 ± 0.15, 2.33 ± 0.29, and 2.67 ± 0.33, respectively (*P* < 0.0001; Fig. 5C), showing increasing c-MET expression as lesions progress from atypia through to HGD. After ROI analysis of the average radiant efficiency values [(p/second/cm^2^/sr)/(μW/cm^2^)] in each group, the signal from lesion versus background in low-, intermediate-, and high-score groups was 34.5 ± 2.70 × 10^7^ versus 22.3 ± 1.68 × 10^7^ (*P* = 0.0015), 91.49 ± 8.74 × 10^7^ versus 48.23 ± 6.09 × 10^7^ (*P* = 0.0007), and 196.89 ± 13.91 × 10^7^ versus 102.3 ± 15.49 × 10^7^ (*P* = 0.0003), respectively (Fig. 5D). The average *ex vivo* TBR values for low-, intermediate-, and high-score groups were 1.58 ± 0.11, 2.04 ± 0.16, and 2.14 ± 0.19, respectively, and were significantly higher than for the PBS-only control group (1.22 ± 0.09; all *P* < 0.001; independent *t* tests; Fig. 5E).

## Discussion

The main finding of this study is that detection of dysplastic lesions in Barrett’s esophagus is feasible using EMI-137–assisted FME in a murine model that phenocopies Barrett’s esophagus metaplasia and dysplasia. c-MET was selected as the molecular target in this study because marked c-MET immunostaining has been shown in Barrett’s esophagus dysplastic lesions and esophageal adenocarcinoma but absent or only modest immunostaining is observed in NDBE (34). In addition, c-MET is expressed on the cell surface and is therefore accessible to a systemically administered imaging tracer. Next the specificity of EMI-137 for c-MET was confirmed through immunostaining of human esophageal cell lines with different levels of c-MET expression and in a dual xenograft model, and these results were found to be consistent with previously reported data (17). Subsequently, the performance of EMI-137 was tested in the L2-IL1β transgenic mouse model of Barrett’s esophagus and dysplasia. Dysplastic lesions were successfully identified under FME in mice, indicating the translational potential of EMI-137 for dysplasia screening during Barrett’s esophagus surveillance in high-risk human populations such as those with gastroesophageal reflux disease (GERD) and known Barrett’s esophagus.

Whereas WLE is currently regarded as the gold standard to detect dysplasia in patients with Barrett’s esophagus and for subsequent surveillance, several interesting noninvasive or minimally invasive experimental approaches prior to WLE have been reported. These include swallowing esophageal endoscopic capsules (35), collecting exhaled volatile organic compounds (36, 37), or using the cytosponge technique to collect cytology samples for analysis of the TFF3 biomarker that identifies intestinal metaplasia (38). The cytosponge–TFF3 procedure was tested in the primary care setting in a randomized trial of patients with GERD and resulted in about a 10-fold increase in the diagnosis of Barrett’s esophagus compared with patients receiving “usual care” (39). In individuals exhibiting TFF3-positive cellular markers as determined by IHC analysis, subsequent endoscopic examination followed by targeted biopsy is mandated. Within this diagnostic framework, particularly in the context of ongoing surveillance of patients with an established diagnosis of early Barrett’s esophagus, the implementation of advanced endoscopic techniques that enhance the precision of biopsies from the suspicious segments of the metaplastic epithelium is imperative. Acquisition of a FME-guided “red flag” biopsy could augment diagnostic fidelity, thereby streamlining the patient’s transition to definitive therapeutic interventions, including endoscopic mucosal resection or endoscopic submucosal resection. Early detection and timely management of potential neoplastic transformation within Barrett’s esophagus metaplasia becomes more realistic with an FME-driven red flag biopsy approach.

Efforts to increase diagnostic accuracy through FME have investigated several molecular markers such as EGFR, ErbB2 receptor tyrosine kinase 2, VEGF-A, fluorescent PARP1 inhibitor, chemokine receptor 4, heat shock protein 70, and c-MET for early esophageal adenocarcinoma detection (13, 15, 16, 40–43). Nagengast and colleagues (13) developed bevacizumab-800CW, a new near-IR fluorescence tracer directed against VEGF-A for Barrett’s esophagus/esophageal adenocarcinoma detection. In a phase 1 safety study involving 14 patients with known Barrett’s esophagus, the delivery of bevacizumab-CW800 to the esophageal mucosa, either by i.v. injection (4.5 mg) or topical spray (100 μg/mL per cm Barrett’s esophagus), contributed to a 33% increase in the detection of dysplastic tissue. The TBR was 2.75 following systemic administration and 4.30 following topical application. However, whereas antibody-based tracers such as bevacizumab are associated with high specificity for the epitope, their manufacture can be challenging and, if given intravenously, their long circulation time may require patients to make return visits for endoscopy after protracted intervals following tracer administration, which may impede clinical adoption.

In 2021, Chen and colleagues (40) reported the use of two heptapeptides (Cy5-labeled QRHKPRE, EGFR-specific, and IRDye800-labeled KSPNPRF, ErbB2-specific) given simultaneously during FME to visualize Barrett’s esophagus (NCT03589443). By combining two optical tracers, these investigators obtained accuracy, sensitivity, and specificity of 91%, 92%, and 89%, respectively, after applying a leave-one-out cross-validation strategy. Nevertheless, the TBR of these tracers was less than 1.4 for NDBE and LGD and was less than 1.7 for both heptapeptides in HGD and esophageal adenocarcinoma, suggesting that targeting EGFR and ErbB2 may not provide optimal dysplasia or esophageal adenocarcinoma detection. In a recent study, Liang and colleagues (42) analyzed the mRNA expression level of *MET* and *EGFR* in esophageal adenocarcinoma by interrogating The Cancer Genome Atlas database, showing that *MET* mRNA is overexpressed in esophageal adenocarcinoma. Immunostaining of 89 esophageal adenocarcinoma samples showed that 58.06% overexpressed c-MET, whereas EGFR overexpression was noted in 27.42%. These data are consistent with the level of c-MET expression noted in the current study (using GEO databases), strengthening the argument for the use of c-MET as a molecular target in early dysplasia detection.

EMI-137 FME was piloted in patients with a range of esophageal pathologies in a phase 1 feasibility and safety study reported by de Jongh and colleagues (12). Fifteen patients with known Barrett’s esophagus-associated dysplasia or esophageal adenocarcinoma underwent EMI-137 FME after either i.v. (0.13 or 0.09 mg/kg) or topical (200 μg/cm) administration of EMI-137 and immediately before scheduled endoscopic mucosal resection. Nineteen lesions were identified on final pathology, of which two were benign. FME was not possible for technical reasons in one patient. Of the remaining 16 lesions, the majority were esophageal adenocarcinoma (*n* = 6) or HGD (*n* = 6) and the remainder showed LGD (*n* = 4). The reported FME detection rate was 89%, and, using surrounding squamous epithelium as background, a TBR of 2.43 ± 0.64 was reported in the cohort receiving EMI-137 0.13 mg/kg intravenously. Importantly, as all the enrolled patients had previously received a diagnosis of Barrett’s esophagus–associated dysplasia or esophageal adenocarcinoma, this study did not address whether EMI-137 could serve as a viable screening test for early dysplasia and esophageal adenocarcinoma detection among new patients presenting with symptoms of GERD in the primary care setting or in general gastroenterology clinics. In this context, a prospective controlled clinical trial would be required to test the feasibility of EMI-137. The purpose of the current preclinical study, therefore, was to gather preclinical data to explore the performance of EMI-137 FME in a murine model that mimics early human Barrett’s esophagus to inform the design of future clinical trials.

It is important to note that the time interval between optical tracer injection and FME image acquisition is critical for optimal image quality and maximum TBR. Based on the results obtained from the dual xenograft model, the strongest fluorescence signal in the OE33 xenograft was at 1 hour p.i. of EMI-137, and the highest TBR was observed at 2 hours p.i. Hence, the 2-hour timepoint was selected for the subsequent FME study. At the 2-hour timepoint, FME of L2-IL1β mice successfully identified dysplastic lesions located at the squamocolumnar junction and EGJ of the stomach with a TBR of 1.82 and 1.88 in the intermediate- and high-score groups of mice, respectively. Likewise, *ex vivo* fluorescence imaging of the esophagus and stomach showed that the TBR reached 2-fold in both the intermediate- and high-score groups.

In previous clinical safety studies, the administered amount of EMI-137 ranged from 0.02 to 0.18 mg/kg intravenously (17). In the current study, the amount of EMI-137 used in the dual xenograft experiment was 0.25 mg/kg (5 μg/mouse), of which the human equivalent dose (HED) is 0.02 mg/kg (calculated as the animal dose × the *K*_*m*_ ratio; ref. 44). The safety dose of EMI-137 in humans is reported to be 0.36 mg/kg, which is equivalent to 4.43 mg/kg in mouse. Therefore, the equivalent amount used to generate clear images in this study would not be expected to cause adverse effects in humans (17, 44). In addition, a greater amount of EMI-137 was given in the L2-IL1β model compared with the dual xenograft model to maximize the Cy5** signal using the available preclinical FME system, which is limited to an excitation wavelength of 680 nm (see Supplementary Material S1). The mass amount of EMI-137 used in the FME study was 2.5 mg/kg (50 μg per mouse) to simulate clinical conditions and accommodate the FME system. No acute adverse effects were observed during any of the *in vivo* experiments, confirming the short-term *in vivo* biosafety of EMI-137 (17). Also, it may be possible to reduce the injected amount required for clear images by increasing the sensitivity of the FME system, optimizing the excitation power density, and using an optimal excitation wavelength in subsequent clinical studies.

The results of this preclinical study indicate the potential of EMI-137 imaging in screening for Barrett’s esophagus–associated dysplastic lesions and esophageal adenocarcinoma and in subsequently allowing the “red flag” biopsy of areas highlighted as abnormal by FME rather than random biopsies. However, some limitations of the study should be noted. It is challenging to interpret FME intensity levels using the available preclinical endoscopy set-up, as it is not possible to control the working distance and viewing angle, factors that markedly affect the detection of the emitted fluorescence in mice, in which the diameter of the esophagus is less than 2 mm. Even so, areas of dysplasia were detectable using FME (Fig. 4A). In comparison to FME, IVIS imaging operates in a highly controlled environment, making it possible to provide much more consistent fluorescence intensity measurements. These differences between the two techniques account for the difference in the plots shown in Figs. 4B and 5C. Importantly, the TBR values derived from FME (Fig. 4C) and IVIS imaging (Fig. 5C) are very similar despite the different imaging approaches and the expected differences in the corresponding fluorescence intensities due to different acquisition parameters (i.e., working distance, viewing angle, and signal and background region definition).

Only a single mass amount of EMI-137 was administered to animals in the dual xenograft and L2-IL1β transgenic mouse experiments. It is possible that a lower amount of EMI-137 would be sufficient for preclinical FME in these models, and this requires further investigation. The available preclinical FME system is restricted to three excitation wavelengths (462, 680, and 750 nm). Therefore, 680 nm was selected for FME in the L2-IL1β model, whereas the emission wavelength of Cy5** is 650 nm. This necessitated administration of 2.5 mg/kg of EMI-137, which could potentially be reduced if a more adjustable endoscopy system becomes available. Lastly, we performed FME in L2-IL1β mice at a single time, 2 hours after EMI-137 administration. Further investigation is needed to determine the optimal tracer administration to imaging time, and whether a later timepoint FME could provide a higher signal to background value is still uncertain.

### Conclusion

EMI-137 accumulates in dysplastic lesions in Barrett’s esophagus and in c-MET–positive esophageal adenocarcinoma. We conclude that EMI-137 has translational potential as an optical real-time tracer for Barrett’s esophagus screening, surveillance, and detection of early dysplasia and esophageal adenocarcinoma. Of note, the dependency of the increase in EMI-137 signal on the stage of dysplasia (low vs. high) could not be fully analyzed in the L2-IL1β mouse model because of the limitations of preclinical FME technology. However, the detection of early dysplasia within an area of Barrett’s esophagus is the highest clinical priority, as HGD is already usually visible using WLE. Thus, the finding that early dysplasia, as present in the L2-IL1β mouse model, is detectable by c-MET–targeted FME is an important outcome of this research. Future clinical trials are needed to explore the optimal dose, route, and image acquisition window, clinical benefits, and cost-effectiveness of EMI-137 in clinical FME.

## Supplementary Material

Supplementary Information S1 Supplementary Information S1. Details of the Fluorescence Molecular Endoscopy (FME) System; Representative videos with screenshot of FME in L2-IL1b transgenic mice

Supplementary Table S1 Supplementary Table S1. Tissue Microarray Metadata (merged data from 4 TMAs used in the study): Correlation between c-MET expression status and patient characteristics and H-score.

Supplementary Table S2 Supplementary Table S2. Tissue Microarray Metadata (merged data from 4 TMAs used in the study): Impact of stage, T-stage, N-stage, and tumor grade on c-MET positivity and H-score of EAC samples.

Supplementary Video S1 Supplementary Video S1. High lesion score mouse: visible bulging lesion with overlaying fluorescence signals as alpha blending in the green channel of the color RGB image. Left, white light imaging; middle, fluorescence imaging; right, overlay imaging.

Supplementary Video S2 Supplementary Video S2. Low lesion score mouse: low to non-fluorescence was detected in normal, flat cardia whereas significant fluorescence was observed in protruding lesion of gastric cardia. Left, white light imaging; middle, fluorescence imaging; right, overlay imaging.

Supplementary Figure S1 Supplementary Figure S1. c-MET immunostaining of FLO-1 and OE33 cells.

Supplementary Figure S2 Supplementary Figure S2. Establishment of a dual xenograft mouse model for testing EMI-137.

Supplementary Figure S3 Supplementary Figure S3. Representative time course fluorescence imaging of the dual xenograft mouse model after EMI-137 injection.

Supplementary Figure S4 Supplementary Figure S4. Fluorescence molecular endoscopy study schema.

Supplementary Figure S5 Supplementary Figure S5. Gross anatomy of mouse stomach.

## References

[1] Uhlenhopp DJ , Then EO, Sunkara T, Gaduputi V. Epidemiology of esophageal cancer: update in global trends, etiology and risk factors. Clin J Gastroenterol 2020;13:1010–21. 32965635 10.1007/s12328-020-01237-x

[2] Sung H , Ferlay J, Siegel RL, Laversanne M, Soerjomataram I, Jemal A, . Global cancer statistics 2020: GLOBOCAN estimates of incidence and mortality worldwide for 36 cancers in 185 countries. CA Cancer J Clin 2021;71:209–49. 33538338 10.3322/caac.21660

[3] Njei B , McCarty TR, Birk JW. Trends in esophageal cancer survival in United States adults from 1973 to 2009: a SEER database analysis. J Gastroenterol Hepatol 2016;31:1141–6. 26749521 10.1111/jgh.13289 PMC4885788

[4] Domper Arnal MJ , Arenas ÁF, Arbeloa ÁL. Esophageal cancer: risk factors, screening and endoscopic treatment in Western and Eastern countries. World J Gastroenterol 2015;21:7933–43. 26185366 10.3748/wjg.v21.i26.7933 PMC4499337

[5] Visrodia K , Singh S, Krishnamoorthi R, Ahlquist DA, Wang KK, Iyer PG, . Magnitude of missed esophageal adenocarcinoma after Barrett's esophagus diagnosis: a systematic review and meta-analysis. Gastroenterology 2016;150:599–607.e7. 26619962 10.1053/j.gastro.2015.11.040 PMC4919075

[6] Hvid-Jensen F , Pedersen L, Drewes AM, Sørensen HT, Funch-Jensen P. Incidence of adenocarcinoma among patients with Barrett's esophagus. N Engl J Med 2011;365:1375–83. 21995385 10.1056/NEJMoa1103042

[7] Que J , Garman KS, Souza RF, Spechler SJ. Pathogenesis and cells of origin of Barrett's esophagus. Gastroenterology 2019;157:349–64.e1. 31082367 10.1053/j.gastro.2019.03.072 PMC6650338

[8] Sharma P , Shaheen NJ, Katzka D, Bergman J. AGA clinical practice update on endoscopic treatment of Barrett's esophagus with dysplasia and/or early cancer: expert review. Gastroenterology 2020;158:760–9. 31730766 10.1053/j.gastro.2019.09.051

[9] Sharma P , Hawes RH, Bansal A, Gupta N, Curvers W, Rastogi A, . Standard endoscopy with random biopsies versus narrow band imaging targeted biopsies in Barrett's oesophagus: a prospective, international, randomised controlled trial. Gut 2013;62:15–21. 22315471 10.1136/gutjnl-2011-300962

[10] Kara MA , Ennahachi M, Fockens P, ten Kate FJ, Bergman JJ. Detection and classification of the mucosal and vascular patterns (mucosal morphology) in Barrett's esophagus by using narrow band imaging. Gastrointest Endosc 2006;64:155–66. 16860062 10.1016/j.gie.2005.11.049

[11] Yamamoto K , Ohnishi S, Mizushima T, Kodaira J, Ono M, Hatanaka Y, . Detection of early adenocarcinoma of the esophagogastric junction by spraying an enzyme-activatable fluorescent probe targeting dipeptidyl peptidase-IV. BMC Cancer 2020;20:64. 31992267 10.1186/s12885-020-6537-9 PMC6988364

[12] de Jongh SJ , Voskuil FJ, Schmidt I, Karrenbeld A, Kats-Ugurlu G, Meersma GJ, . C-Met targeted fluorescence molecular endoscopy in Barrett's esophagus patients and identification of outcome parameters for phase-I studies. Theranostics 2020;10:5357–67. 32373217 10.7150/thno.42224 PMC7196285

[13] Nagengast WB , Hartmans E, Garcia-Allende PB, Peters FTM, Linssen MD, Koch M, . Near-infrared fluorescence molecular endoscopy detects dysplastic oesophageal lesions using topical and systemic tracer of vascular endothelial growth factor A. Gut 2019;68:7–10. 29247063 10.1136/gutjnl-2017-314953 PMC6839834

[14] Neves AA , Di Pietro M, O’Donovan M, Waterhouse DJ, Bohndiek SE, Brindle KM, . Detection of early neoplasia in Barrett's esophagus using lectin-based near-infrared imaging: an ex vivo study on human tissue. Endoscopy 2018;50:618–25. 29342490 10.1055/s-0043-124080 PMC6193410

[15] Marcazzan S , Braz Carvalho MJ, Konrad M, Strangmann J, Tenditnaya A, Baumeister T, . CXCR4 peptide-based fluorescence endoscopy in a mouse model of Barrett's esophagus. EJNMMI Res 2022;12:2. 35006394 10.1186/s13550-021-00875-7 PMC8748556

[16] Fang H-Y , Stangl S, Marcazzan S, Carvalho MJB, Baumeister T, Anand A, . Targeted Hsp70 fluorescence molecular endoscopy detects dysplasia in Barrett's esophagus. Eur J Nucl Med Mol Imaging 2022;49:2049–63. 34882260 10.1007/s00259-021-05582-y PMC9016004

[17] Burggraaf J , Kamerling IM, Gordon PB, Schrier L, de Kam ML, Kales AJ, . Detection of colorectal polyps in humans using an intravenously administered fluorescent peptide targeted against c-Met. Nat Med 2015;21:955–61. 26168295 10.1038/nm.3641

[18] Vries HM , Bekers E, van Oosterom MN, Karakullukcu MB, van HG, Poel D, . c-MET receptor-targeted fluorescence on the road to image-guided surgery in penile squamous cell carcinoma patients. J Nucl Med 2022;63:51–6. 33990404 10.2967/jnumed.120.261864 PMC8717176

[19] Metman MJH , Jonker PKC, Sondorp LHJ, van Hemel BM, Sywak MS, Gill AJ, . MET-receptor targeted fluorescent imaging and spectroscopy to detect multifocal papillary thyroid cancer. Eur J Nucl Med Mol Imaging 2024;51:2384–94. 38017325 10.1007/s00259-023-06525-5 PMC11178647

[20] Atreya R , Goetz M. Molecular imaging in gastroenterology. Nat Rev Gastroenterol Hepatol 2013;10:704–12. 23856892 10.1038/nrgastro.2013.125

[21] Jonker PKC , Metman MJH, Sondorp LHJ, Sywak MS, Gill AJ, Jansen L, . Intraoperative MET-receptor targeted fluorescent imaging and spectroscopy for lymph node detection in papillary thyroid cancer: novel diagnostic tools for more selective central lymph node compartment dissection. Eur J Nucl Med Mol Imaging 2022;49:3557–70. 35389070 10.1007/s00259-022-05763-3 PMC9308606

[22] Buckle T , van Alphen M, van Oosterom MN, van Beurden F, Heimburger N, van der Wal JE, . Translation of c-Met targeted image-guided surgery solutions in oral cavity cancer-initial proof of concept data. Cancers (Basel) 2021;13:2674. 34071623 10.3390/cancers13112674 PMC8198422

[23] de Jongh SJ , Vrouwe JPM, Voskuil FJ, Schmidt I, Westerhof J, Koornstra JJ, . The optimal imaging window for dysplastic colorectal polyp detection using c-Met-targeted fluorescence molecular endoscopy. J Nucl Med 2020;61:1435–41. 32198312 10.2967/jnumed.119.238790

[24] Esfahani SA , Heidari P, Kim SA, Ogino S, Mahmood U. Optical imaging of mesenchymal epithelial transition factor (MET) for enhanced detection and characterization of primary and metastatic hepatic tumors. Theranostics 2016;6:2028–38. 27698938 10.7150/thno.15718 PMC5039678

[25] Armstrong GR , Khot MI, Portal C, West NP, Perry SL, Maisey TI, . A novel fluorescent c-Met targeted imaging agent for intra-operative colonic tumour mapping: translation from the laboratory into a clinical trial. Surg Oncol 2022;40:101679. 34839199 10.1016/j.suronc.2021.101679

[26] Wang Q , Ma C, Kemmner W. Wdr66 is a novel marker for risk stratification and involved in epithelial-mesenchymal transition of esophageal squamous cell carcinoma. BMC Cancer 2013;13:137. 23514407 10.1186/1471-2407-13-137 PMC3610187

[27] Kim SM , Park Y-Y, Park ES, Cho JY, Izzo JG, Zhang D, . Prognostic biomarkers for esophageal adenocarcinoma identified by analysis of tumor transcriptome. PLoS One 2010;5:e15074. 21152079 10.1371/journal.pone.0015074 PMC2994829

[28] Barrett T , Wilhite SE, Ledoux P, Evangelista C, Kim IF, Tomashevsky M, . NCBI GEO: archive for functional genomics data sets–update. Nucleic Acids Res 2013;41:D991–5. 23193258 10.1093/nar/gks1193 PMC3531084

[29] Bankhead P , Loughrey MB, Fernández JA, Dombrowski Y, McArt DG, Dunne PD, . QuPath: open source software for digital pathology image analysis. Sci Rep 2017;7:16878. 29203879 10.1038/s41598-017-17204-5 PMC5715110

[30] Quante M , Bhagat G, Abrams JA, Marache F, Good P, Lee MD, . Bile acid and inflammation activate gastric cardia stem cells in a mouse model of Barrett-like metaplasia. Cancer Cell 2012;21:36–51. 22264787 10.1016/j.ccr.2011.12.004 PMC3266546

[31] Münch NS , Fang H-Y, Ingermann J, Maurer HC, Anand A, Kellner V, . High-fat diet accelerates carcinogenesis in a mouse model of Barrett's esophagus via interleukin 8 and alterations to the gut microbiome. Gastroenterology 2019;157:492–506.e2. 30998992 10.1053/j.gastro.2019.04.013 PMC6662596

[32] Fox JG , Beck P, Dangler CA, Whary MT, Wang TC, Shi HN, . Concurrent enteric helminth infection modulates inflammation and gastric immune responses and reduces helicobacter-induced gastric atrophy. Nat Med 2000;6:536–42. 10802709 10.1038/75015

[33] Glatz J , Symvoulidis P, Garcia-Allende PB, Ntziachristos V. Robust overlay schemes for the fusion of fluorescence and color channels in biological imaging. J Biomed Opt 2014;19:040501. 24695844 10.1117/1.JBO.19.4.040501

[34] Herrera LJ , El-Hefnawy T, Queiroz de Oliveira PE, Raja S, Finkelstein S, Gooding W, . The HGF receptor c-Met is overexpressed in esophageal adenocarcinoma. Neoplasia 2005;7:75–84. 15720819 10.1593/neo.04367 PMC1490312

[35] Duvvuri A , Desai M, Vennelaganti S, Higbee A, Gorrepati VS, Dasari C, . Diagnostic accuracy of a novel third generation esophageal capsule as a non-invasive detection method for Barrett's esophagus: a pilot study. J Gastroenterol Hepatol 2021;36:1222–5. 32996655 10.1111/jgh.15283

[36] Peters Y , Schrauwen RWM, Tan AC, Bogers SK, de Jong B, Siersema PD. Detection of Barrett's oesophagus through exhaled breath using an electronic nose device. Gut 2020;69:1169–72. 32098798 10.1136/gutjnl-2019-320273

[37] Kumar S , Huang J, Abbassi-Ghadi N, Mackenzie HA, Veselkov KA, Hoare JM, . Mass spectrometric analysis of exhaled breath for the identification of volatile organic compound biomarkers in esophageal and gastric adenocarcinoma. Ann Surg 2015;262:981–90. 25575255 10.1097/SLA.0000000000001101

[38] Offman J , Muldrew B, O’Donovan M, Debiram-Beecham I, Pesola F, Kaimi I, . Barrett's oESophagus trial 3 (BEST3): study protocol for a randomised controlled trial comparing the cytosponge-TFF3 test with usual care to facilitate the diagnosis of oesophageal pre-cancer in primary care patients with chronic acid reflux. BMC Cancer 2018;18:784. 30075763 10.1186/s12885-018-4664-3 PMC6091067

[39] Fitzgerald RC , di Pietro M, O’Donovan M, Maroni R, Muldrew B, Debiram-Beecham I, . Cytosponge-trefoil factor 3 versus usual care to identify Barrett's oesophagus in a primary care setting: a multicentre, pragmatic, randomised controlled trial. Lancet 2020;396:333–44. 32738955 10.1016/S0140-6736(20)31099-0 PMC7408501

[40] Chen J , Jiang Y, Chang T-S, Joshi B, Zhou J, Rubenstein JH, . Multiplexed endoscopic imaging of Barrett's neoplasia using targeted fluorescent heptapeptides in a phase 1 proof-of-concept study. Gut 2021;70:1010–3. 33028666 10.1136/gutjnl-2020-322945 PMC8108279

[41] Gabriëls RY , van Heijst LE, Hooghiemstra WTR, van der Waaij AM, Kats-Ugurlu G, Karrenbeld A, . Detection of early esophageal neoplastic Barrett lesions with quantified fluorescence molecular endoscopy using cetuximab-800CW. J Nucl Med 2023;64:803–8. 36604181 10.2967/jnumed.122.264656

[42] Liang M , Wang L, Xiao Y, Yang M, Mei C, Zhang Y, . Preclinical evaluation of a novel EGFR&c-Met bispecific near infrared probe for visualization of esophageal cancer and metastatic lymph nodes. Eur J Nucl Med Mol Imaging 2023;50:2787–801. 37145165 10.1007/s00259-023-06250-z

[43] Marcazzan S , Braz Carvalho MJ, Nguyen NT, Strangmann J, Slotta-Huspenina J, Tenditnaya A, . PARP1-targeted fluorescence molecular endoscopy as novel tool for early detection of esophageal dysplasia and adenocarcinoma. J Exp Clin Cancer Res 2024;43:53. 38383387 10.1186/s13046-024-02963-7 PMC10880256

[44] Nair AB , Jacob S. A simple practice guide for dose conversion between animals and human. J Basic Clin Pharm 2016;7:27–31. 27057123 10.4103/0976-0105.177703 PMC4804402

