# Supplementary material for: Targeting c-MET for Endoscopic Detection of Dysplastic Lesions within Barrett’s Esophagus Using EMI-137 Fluorescence Imaging
Source: Clin Cancer Res. 2024 Nov 8;31(1):98–109. doi: 10.1158/1078-0432.CCR-24-1522 (PMC11701434; doi:10.1158/1078-0432.CCR-24-1522)
Supplement: Supplementary Information S1 — Details of the Fluorescence Molecular Endoscopy (FME) System; Representative videos with screenshot of FME in L2-IL1b transgenic mice [file ccr-24-1522_supplementary_information_s1_suppsi1.pdf]

Supplementary Information

SI–1. Details of the Fluorescence Molecular Endoscopy (FME) System

FME was enabled by a custom-made imaging system developed by Helmholtz Munich and modified for mouse endoscopy. Briefly, a flexible fiberscope (i.e., 0.8-mm outer diameter, 6000 pixels, Microendo-Fiberskop, SCHÖLLY FIBEROPTIC GMBH) was coupled to a highly sensitive electron-multiplying charge-couple device (EMCCD) (DU-897U-CS0, Andor Technology) through a 685 nm filter (FF01-685-LP, Semrock). A 685 nm dichroic mirror (FF685-Di02, Semrock) was used to allow color imaging through a high-resolution color charge-coupled device (CCD) camera (BFS-U3-23S3C-C, FLIR Systems, Inc.) with a 665 short-pass filter (FF01-665/SP-25, Semrock) to cut off the excitation source light. Fluorescence was induced through a 680 nm laser diode (FLX-680-1000M-150, Frankfurt Laser Company) controlled by the PRO 8000 rack system (LDM90/M, LDC8020, TED8040, and PRO8000-4, Thorlabs), while the illumination for color imaging was a 250-W halogen lamp (KL-2500 LCD, Schott AG) filtered with a 665 nm short-pass filter (FF01-665/SP-25, Semrock) to match the detection spectrum of the color camera. The excitation source power at the distal end of the flexible fiberscope complies with the American National Standards Institute (ANSI) and the European Standards (EN) limits for the maximum permissive exposure in skin (200 mW/cm^2 measured at distance < 2 mm). Both light sources are coupled into a multimode bifurcated fiber-bundle (Leoni FiberOptics) connected to the light guide of the fiberscope. The control of the system,

data acquisition, and real-time visualization of co-registered fluorescence and color images were implemented through a custom-made C++ software developed by the Helmholtz Munich.

SI–2. Representative videos with screenshot of FME in L2-IL1β transgenic mice

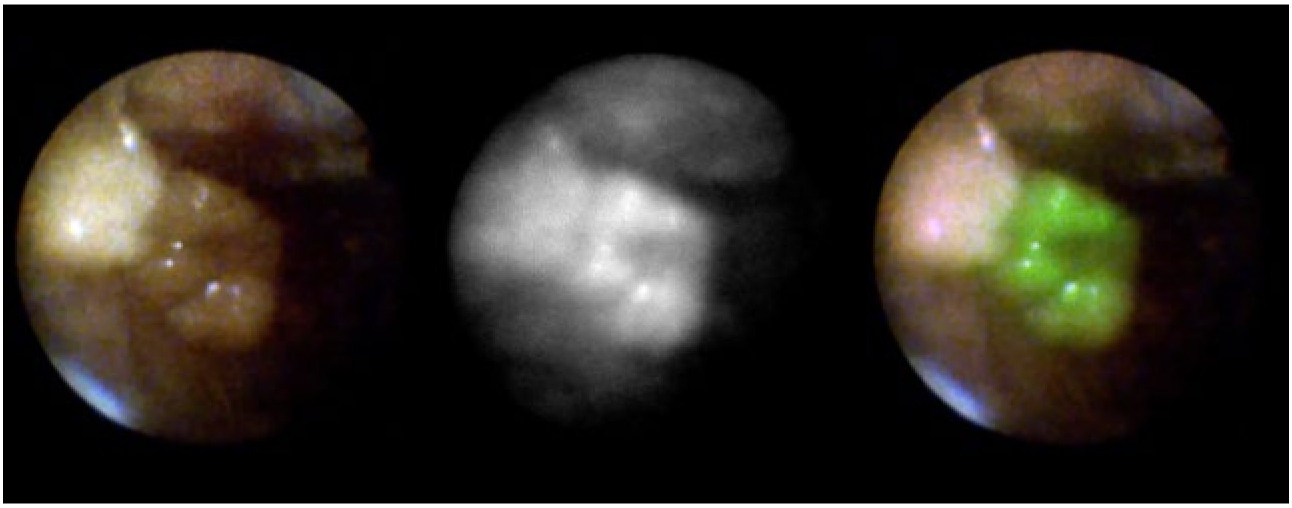

Supplementary Video S1. High lesion score mouse: visible bulging lesion with overlaying fluorescence signals as alpha blending in the green channel of the color RGB image. Left, white light imaging; middle, fluorescence imaging; right, overlay imaging.

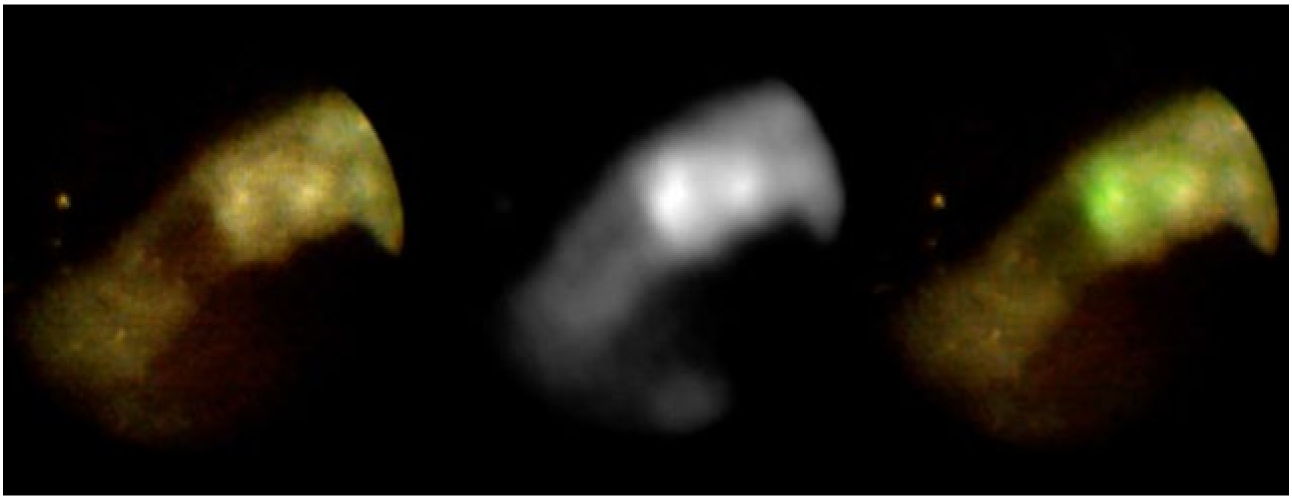

Supplementary Video S2. Low lesion score mouse: low to non-fluorescence was detected in normal, flat cardia whereas significant fluorescence was observed in protruding lesion of gastric cardia. Left, white light imaging; middle, fluorescence imaging; right, overlay imaging.
