# Supplementary material for: Targeting c-MET for Endoscopic Detection of Dysplastic Lesions within Barrett’s Esophagus Using EMI-137 Fluorescence Imaging
Source: Clin Cancer Res. 2024 Nov 8;31(1):98–109. doi: 10.1158/1078-0432.CCR-24-1522 (PMC11701434; doi:10.1158/1078-0432.CCR-24-1522)
Supplement: Supplementary Table S1 — Tissue Microarray Metadata (merged data from 4 TMAs used in the study): Correlation between c-MET expression status and patient characteristics and H-score. [file ccr-24-1522_supplementary_table_s1_suppst1.docx]

**Supplementary Table 1. Tissue Microarray Metadata (merged data from 4 TMAs used in the study): Correlation between c-MET expression status and patient characteristics and H-score**

|  | NSE  (n = 29) | Inflammation  (n = 50) | Hyperplasia  (n = 68) | EAC  (n = 104) | *p value* |
| --- | --- | --- | --- | --- | --- |
| Age | 40.03 ± 2.95 | 56.48 ± 1.05 | 58.35 ± 1.07 | 58.80 ± 0.96 | < 0.001 |
| Gender (M:F) | 20:9 | 31:19 | 40:28 | 76:28 | 0.228 |
| Positivity (%) | 54.54 ± 3.42 | 69.70 ± 3.01 | 76.08 ± 2.46 | 94.51 ± 1.05 | < 0.001 |
| H-score | 82.51 ± 6.41 | 92.00 ± 5.45 | 112.50 ± 6.20 | 205.80 ± 6.74 | < 0.001 |
