# Supplementary material for: Targeting c-MET for Endoscopic Detection of Dysplastic Lesions within Barrett’s Esophagus Using EMI-137 Fluorescence Imaging
Source: Clin Cancer Res. 2024 Nov 8;31(1):98–109. doi: 10.1158/1078-0432.CCR-24-1522 (PMC11701434; doi:10.1158/1078-0432.CCR-24-1522)
Supplement: Supplementary Table S2 — Tissue Microarray Metadata (merged data from 4 TMAs used in the study): Impact of stage, T-stage, N-stage, and tumor grade on c-MET positivity and H-score of EAC samples. [file ccr-24-1522_supplementary_table_s2_suppst2.docx]

**Supplementary Table 2. Tissue Microarray Metadata (merged data from 4 TMAs used in the study): Impact of stage, T-stage, N-stage, and tumor grade on c-MET positivity and H-score of EAC samples**

|  | c-MET  Positivity (%) | *p value* | c-MET  H-score | *p value* |
| --- | --- | --- | --- | --- |
| Stages |  |  |  |  |
| 0 (n=7) | 86.06 ± 8.08 | 0.067 | 158.11 ± 26.9 | 0.003 |
| II (n=48) | 96.83 ± 0.96 |  | 220.98 ± 9.18 |  |
| III (n=31) | 93.71 ± 2.14 |  | 217.83 ± 12.5 |  |
| Unknown | 93.00 ± 2.57 |  | 162.94 ± 6.74 |  |
| T-stage |  |  |  |  |
| Tis (n=7) | 86.06 ± 8.08 | 0.153 | 158.11 ± 26.9 | 0.005 |
| T2 (n=20) | 95.97 ± 2.02 |  | 212.81 ± 14.8 |  |
| T3 (n=48) | 96.20 ± 1.02 |  | 223.85 ± 9.13 |  |
| T4 (n=11) | 92.36 ± 4.74 |  | 214.44 ± 23.7 |  |
| Unknown T | 93.00 ± 2.57 |  | 162.94 ± 13.6 |  |
| N-stage |  |  |  |  |
| N0 (n=56) | 94.21 ± 1.60 | 0.623 | 206.17 ± 9.41 | 0.003 |
| N1 (n=30) | 95.98 ± 1.48 |  | 230.69 ± 11.1 |  |
| Unknown N | 93.00 ± 2.57 |  | 162.94 ± 13.6 |  |
| Tumor grade |  |  |  |  |
| G1 (n=9) | 87.16 ± 6.26 | 0.087 | 160.44 ± 21.2 | 0.100 |
| G2 (n=38) | 93.35 ± 2.03 |  | 199.00 ± 11.4 |  |
| G3 (n=54) | 96.48 ± 0.94 |  | 218.60 ± 8.99 |  |
| Unknown G | 95.86 ± 2.11 |  | 196.31 ± 6.74 |  |
