# Supplementary material for: Targeting c-MET for Endoscopic Detection of Dysplastic Lesions within Barrett’s Esophagus Using EMI-137 Fluorescence Imaging
Source: Clin Cancer Res. 2024 Nov 8;31(1):98–109. doi: 10.1158/1078-0432.CCR-24-1522 (PMC11701434; doi:10.1158/1078-0432.CCR-24-1522)
Supplement: Supplementary Figure S1 — c-MET immunostaining of FLO-1 and OE33 cells. [file ccr-24-1522_supplementary_figure_s1_suppsf1.pdf]

Supplementary Data

Figure S1

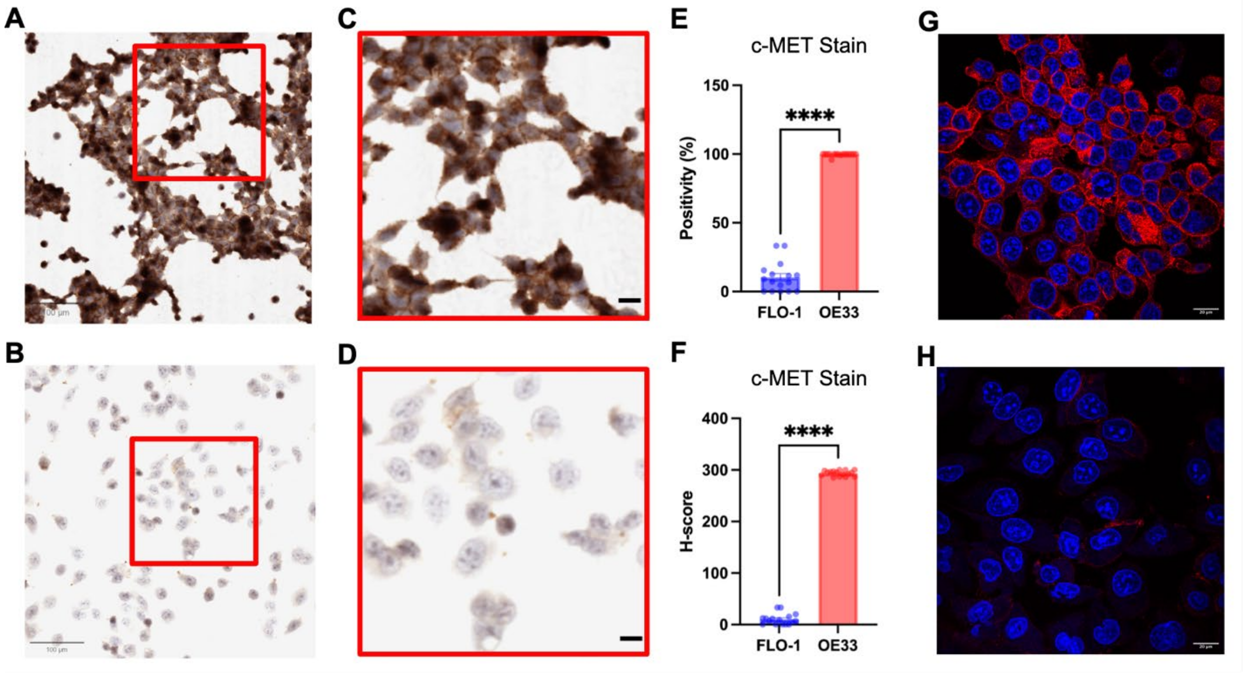

Supplementary Figure S1. c-MET immunostaining of FLO-1 and OE33 cells

For immunohistochemistry, (**A**) OE33 and (**B**) FLO-1 cells were fixed and permeabilized using standard procedures and then incubated with c-MET primary antibody (EP1454Y), followed by biotinylated anti-rabbit secondary IgG, and DAB. More than 90% of FLO-1 cells were negative for c-MET expression (scale bar – 100 µm). High magnification images of the inset (red box) for (**C**) OE33 and (**D**) FLO-1 cells are shown (scale bar - 20 µm). (**E**) The percentage of cells that were c-MET positive was significantly higher in OE33 versus FLO-1 cells. (**F**) c-MET expression was significantly higher in OE33 versus FLO-1 (H-score: 293.5±1.32 *versus* 10.33±2.72). Immunofluorescence microscopy was performed following immunostaining of **(G)** OE33 and (**H)**

FLO-1 cells with primary antibody EP1454Y, and secondary antibody AF594-GAR (invitrogen, Catalog # A-11012). Marked cell surface c-MET expression was observed in OE33 but not FLO-1 cells. **** $p < 0.0001$
