# Supplementary material for: Targeting c-MET for Endoscopic Detection of Dysplastic Lesions within Barrett’s Esophagus Using EMI-137 Fluorescence Imaging
Source: Clin Cancer Res. 2024 Nov 8;31(1):98–109. doi: 10.1158/1078-0432.CCR-24-1522 (PMC11701434; doi:10.1158/1078-0432.CCR-24-1522)
Supplement: Supplementary Figure S2 — Establishment of a dual xenograft mouse model for testing EMI-137. [file ccr-24-1522_supplementary_figure_s2_suppsf2.pdf]

# Figure S2

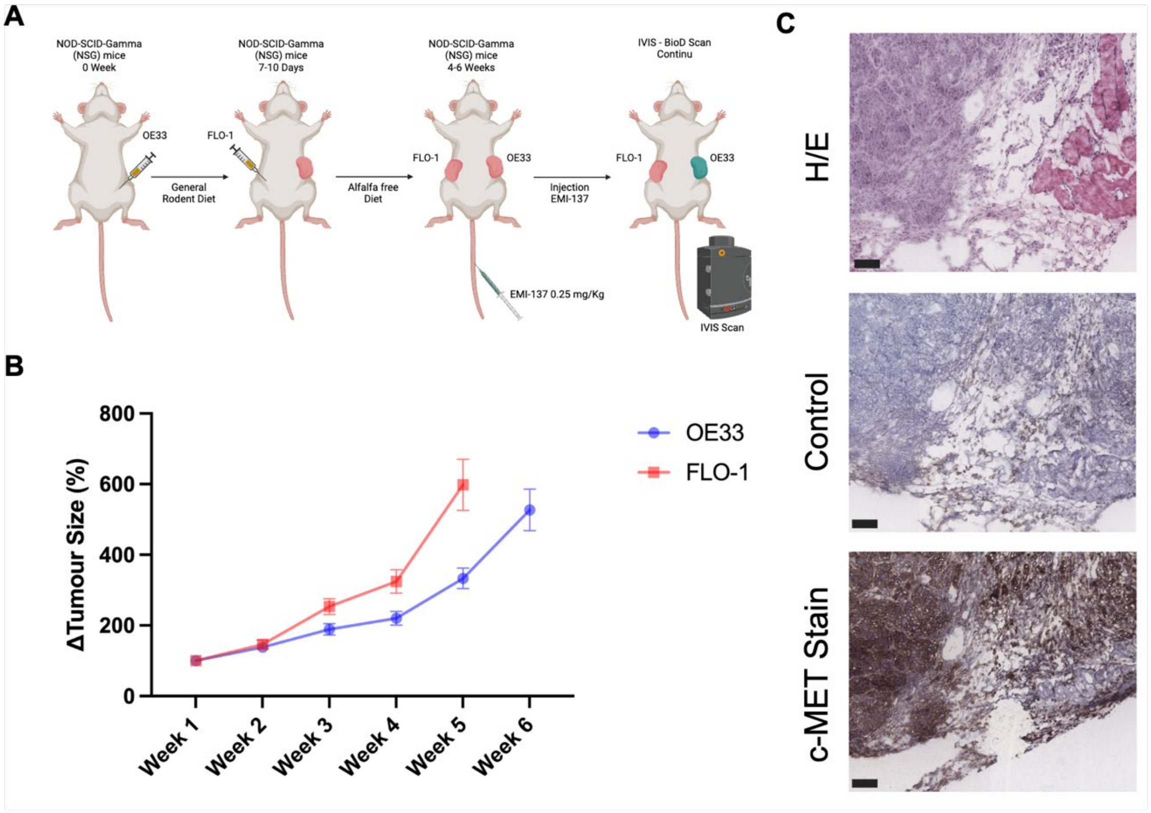


**Supplementary Figure S2. Establishment of a dual xenograft mouse model for testing EMI-137**

**(A)** Schematic diagram of the dual xenograft model with FLO-1 (left) and OE33 (right) tumors. **(B)** The growth rate of FLO-1 tumors is faster than OE33 tumors. **(C)** H/E stain and c-MET immunostaining confirmed that c-MET overexpression persists *in vivo* in OE33 xenografts. Scale bar - 100 µm. (A, Created with BioRender.com).
