# Supplementary material for: Targeting c-MET for Endoscopic Detection of Dysplastic Lesions within Barrett’s Esophagus Using EMI-137 Fluorescence Imaging
Source: Clin Cancer Res. 2024 Nov 8;31(1):98–109. doi: 10.1158/1078-0432.CCR-24-1522 (PMC11701434; doi:10.1158/1078-0432.CCR-24-1522)
Supplement: Supplementary Figure S3 — Representative time course fluorescence imaging of the dual xenograft mouse model after EMI-137 injection. [file ccr-24-1522_supplementary_figure_s3_suppsf3.pdf]

Figure S3

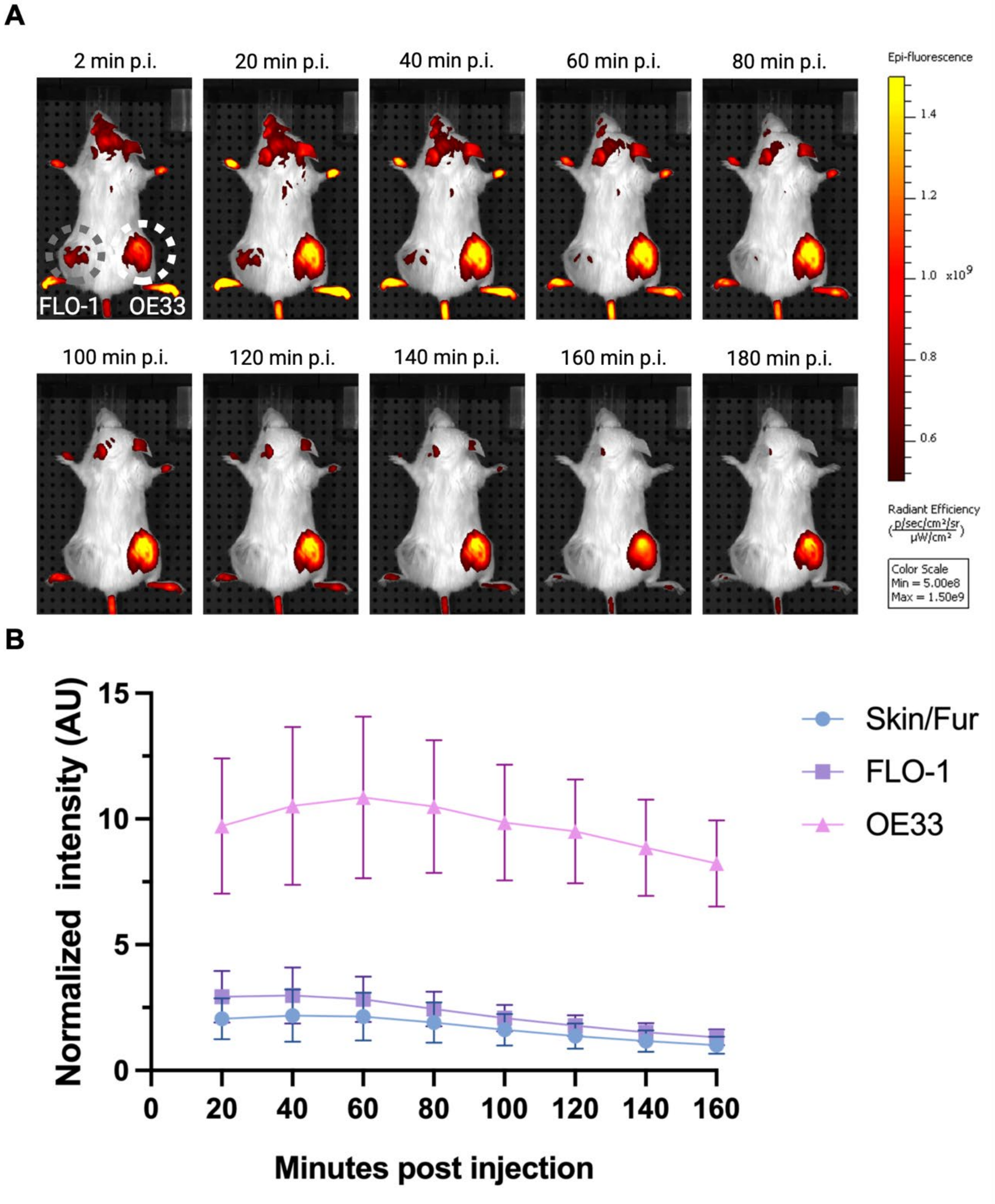


**Supplementary Figure S3. Representative time course fluorescence imaging of the dual xenograft mouse model after EMI-137 injection**

**(A)** Representative IVIS images were captured every 20 minutes p.i. of EMI-137 (0.25 mg/kg), confirming the fluorescence signal was selectively retained in OE33 (c-MET positive, right, white

circle) but not FLO-1 (c-MET negative, left, grey circle) tumors. **(B)** Dynamic change of normalized fluorescence intensities obtained from IVIS system images. Data were normalized to the average intensity of skin/fur at the end of the experiment.
