# Supplementary material for: Targeting c-MET for Endoscopic Detection of Dysplastic Lesions within Barrett’s Esophagus Using EMI-137 Fluorescence Imaging
Source: Clin Cancer Res. 2024 Nov 8;31(1):98–109. doi: 10.1158/1078-0432.CCR-24-1522 (PMC11701434; doi:10.1158/1078-0432.CCR-24-1522)
Supplement: Supplementary Figure S4 — Fluorescence molecular endoscopy study schema. [file ccr-24-1522_supplementary_figure_s4_suppsf4.pdf]

Figure S4

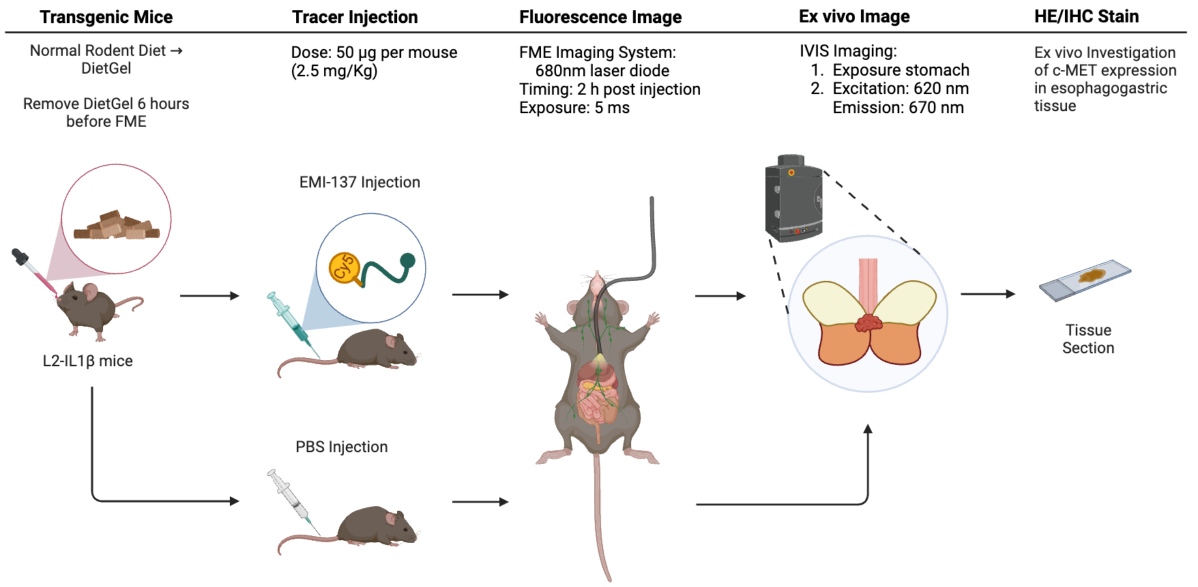

Supplementary Figure S4. Fluorescence molecular endoscopy study schema

Study design of real-time testing of EMI-137 in L2-IL1β transgenic mice. Mice were separated into low, intermediate, and high anticipated tumor score groups based on age. Two hours after i.v. administration of EMI-137, the mice were euthanized. FME and *ex vivo* fluorescence imaging were performed. The dysplasia score of tissues was determined *ex vivo*. HE, hematoxylin and eosin; IHC, immunohistochemistry. (Created with BioRender.com).
