# Supplementary material for: Targeting c-MET for Endoscopic Detection of Dysplastic Lesions within Barrett’s Esophagus Using EMI-137 Fluorescence Imaging
Source: Clin Cancer Res. 2024 Nov 8;31(1):98–109. doi: 10.1158/1078-0432.CCR-24-1522 (PMC11701434; doi:10.1158/1078-0432.CCR-24-1522)
Supplement: Supplementary Figure S5 — Gross anatomy of mouse stomach. [file ccr-24-1522_supplementary_figure_s5_suppsf5.pdf]

Figure S5

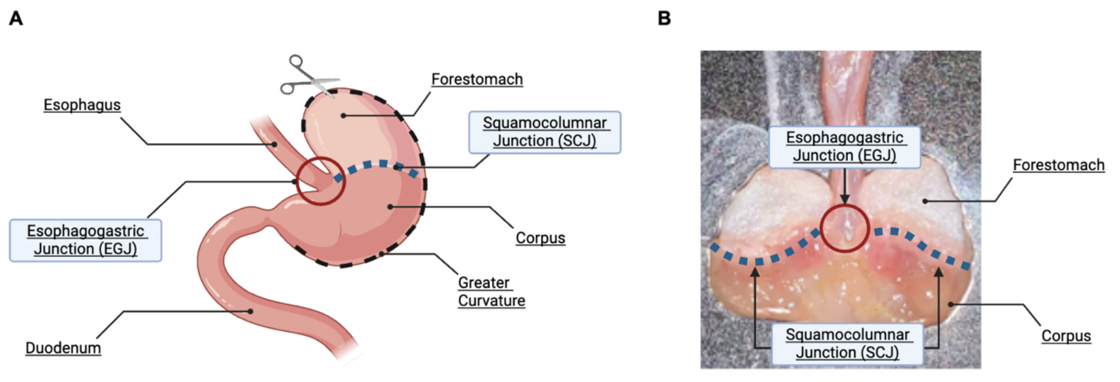

Supplementary Figure S5. Gross anatomy of mouse stomach

(A) Schematic diagram of the mouse stomach depicting the squamocolumnar junction (SCJ, blue dotted line) and esophagogastric junction (EGJ, red circle) of the mouse. **(B)** A stomach cut open along the greater curvature to expose the gastric mucosa. Progression of BE metaplasia appears at SCJ and EGJ of the L2-IL1β mouse stomach. (A-B, Created with BioRender.com).
